# Molecular mechanism of estrogen-mediated neuroprotection in the relief of brain ischemic injury

**DOI:** 10.1186/s12863-018-0630-y

**Published:** 2018-07-20

**Authors:** Jiaxuan He, Ya Gao, Gang Wu, Xiaoming Lei, Yong Zhang, Weikang Pan, Hui Yu

**Affiliations:** 1 Department of Anesthesia, Second Affiliated Hospital of Xi’an Jiaotong University, Xi’an, 710004 China; 2 Department of Pediatric surgery, Second Affiliated Hospital of Xi’an Jiaotong University, No.157, XiWu Road, Xi’an, 710004 China

**Keywords:** Brain ischemic injury, Estrogen, Differentially expressed genes, microRNAs, Pathway enrichment analysis

## Abstract

**Background:**

This study aimed to explore the molecular mechanism of estrogen-mediated neuroprotection in the relief of cerebral ischemic injury. The gene expression profiles were downloaded from Gene Expression Omnibus database, and differentially expressed genes (DEGs) were identified using limma package in R software. Further, DEGs were subjected to Gene Ontology (GO) cluster analysis using online Gene Ontology Enrichment Analysis Software Toolkit and to GO functional enrichment analysis using DAVID software. Using the Gene Set Analysis Toolkit V2, enrichment analysis of Kyoto Encyclopedia of Genes and Genomes pathways was performed. In addition, protein-protein interaction (PPI) network was constructed using STRING database, and submodule analysis of PPI network. Lastly, the significant potential target sites of microRNAs (miRNAs) were predicted using Molecular Signatures Database, and the function analysis of targets of predicted miRNA was also performed using DAVID software.

**Results:**

In total, 321 DEGs were screened in the estrogen-treated sample. The DEGs were mainly associated with intracellular signaling and metabolic pathways, such as calcium channel, calcineurin complex, insulin secretion, low-density lipoprotein reconstruction, and starch or sugar metabolism. In addition, GO enrichment analysis indicated an altered expression of the genes related to starch and sucrose metabolism, retinol metabolism, anti-apoptosis (eg., *BDNF* and *ADAM17*) and response to endogenous stimulus. The constructed PPI network comprised of 243 nodes and 590 interaction pairs, and four submodules were obtained from PPI network. Among the module d, four glutamate receptors as Gria4, Gria3, Grin3a and Grik4 were highlighted. Further, 5 novel potential regulatory miRNAs were also predicted. MIR-338 and MIR520D were closely associated with cell cycle, while the targets of MIR-376A and MIR-376B were only involved in cell soma.

**Conclusions:**

The DEGs in estrogen-treated samples are closely associated with calcium channel, glutamate induced excitotoxicity and anti-apoptotic activity. In addition, some functionally significant DEGs such as *BDNF*, *ADAM17*, *Gria4*, *Gria3*, *Grin3a*, *Grik4, Gys2* and *Ugtla2*, and new miRNAs like MIR-338 and MIR-376A were identified, which may serve as potential therapeutic targets for the effective treatment of cerebral ischemic injury.

**Electronic supplementary material:**

The online version of this article (10.1186/s12863-018-0630-y) contains supplementary material, which is available to authorized users.

## Background

Stroke, the third leading cause of death in the developed countries, has been extensively studied over the past decades [1]. Cerebral ischemia is predominantly caused by the thromboembolic occlusion of the major cerebral artery or its branches leading to a transient or permanent reduction in cerebral blood flow [2]. The pathogenic mechanisms of cerebral ischemic injury occur through a complex interplay of several molecular pathways, including excitotoxicity, peri-infarct depolarizations, inflammation, and apoptosis [3]. As one of the high energy-intensive part, the physiological equilibrium of brain tissue is disrupted and energy supply is cut off. Consequently, the voltage-dependent Ca^2+^ channels are activated [4]. Moreover, energy deprivation and K^+^ and glutamate release can trigger the depolarization of ischemic neurons and glia, and the activation of depolarizations may increase infarct volume and size that has been studied in the rats [5]. In addition to infarct formation, the activation of intracellular second messenger system and excessive production of free radicals can induce the expression of a spectrum of genes involved in the pro-inflammatory response [6].

Because of the high mortality associated with cerebral ischemic injury, new treatment approaches and therapeutic strategies have been widely investigated. Tsai et al. have shown that resveratrol exhibits neuroprotective effect during cerebral ischemic injury through nitric oxide mechanism [7]. Flavonoids extracted from a Scutellaria baicalensis Georgi have been demonstrated to be effective for treatment of cerebral ischemic injury [8]. Moreover, gypenosides, green tea extract, Pueraria extracts, and garlic extracts [9] have been used for treating stroke-induced brain damage and loss of neuronal function. Notably, estrogen has been demonstrated to enhance cognitive function and reduce neurodegenerative risk associated with stroke in postmenopausal women [10]. Physiologically relevant levels of estrogen can significantly reduce infarct volume and protect against neurodegeneration [10]. However, the detailed mechanism by which estrogen mediates these protective effects remains unclear.

Therefore, the present study aimed to explore the molecular mechanism of estrogen-mediated neuroprotection in cerebral ischemic injury by identifying the functions and enriched pathways of differentially expressed genes (DEGs) using bioinformatic analysis of microarray data. Furthermore, a microRNA (miRNA)-binding site enrichment analysis was predicted.

## Methods

### Microarray data collection

Gene microarray data of GSE5315 [11] that included two estrogen-treated samples and two control samples were downloaded from the Gene Expression Omnibus (GEO) database (https://www.ncbi.nlm.nih.gov/geo/). The data were obtained using Affymetrix Rat Genome U34 array set (RG_U34A) GPL85. In the GSE5315, female rats aged 8–10 weeks were ovariectomized and the ovariectomized rats that implanted s.c. with 21-day release pellets containing 25 μg of 17β-estradiol or Placebo were divided into estrogen-treated group and control group, respectively. Then, transient focal cerebral ischemia was induced in the rats from above two groups by intraluminal middle cerebral artery occlusion (MCAO). At 6 and 24 h after MCAO 2 h, rats were decapitated under deep halothane anesthesia, and the brains were quickly removed and frozen (*n* = 6 per group at each time point, was considered as one sample) [11]. In this study, we only extracted and re-analyzed the microarray data of GSE5315 dataset provided by Xu et al., and we didn’t need to conduct the above experiments on rats. Therefore, the animal ethics approval was not needed for the present study.

### *Identification of* DEG

The data were retrieved using GEOquery and processed using limma packages in R software [12]. The preprocessed expression data were obtained using GEOquery package, and normalized intensity data were log_2_ transformed and subjected to further analysis. To identify DEGs between the experimental and control groups, Bayes t-test of Benjamini–Hochberg correction was applied. *p* values of < 0.05 were considered to indicate statistical significance.

### Protein-protein interaction (PPI) network construction and submodule analysis of PPI network

The Search Tool for the Retrieval of Interacting Genes (STRING, https://string-db.org/) [13] database provides information on protein-protein interactions for numerous organisms. The STRING database was applied to predict the PPIs edited by DEGs, and the parameter of combined score > 0.4 was set as the threshold value for choosing significant interactions. Then, the Cytoscape software (http://www.cytoscape.org/) was used to construct the PPI network through visualizing the significant interactions [14]. In addition, the nodes in PPI network were ranked by their connectivity degrees, which correspond to the number of interactions by other proteins. Moreover, submodule analysis is a useful method to divide the PPI network into several modules, in which proteins with similar function tend to cluster together. The MCODE plug-in in Cytoscape was used to conduct the submodule analysis with the threshold value of score ≥ 3.

### Gene ontology (GO) functional enrichment analysis

To interpret the biological function of the DEGs, GO [15] analysis was performed using Gene Ontology Enrichment Analysis Software Toolkit (GOEAST) [16], which is a web-based software toolkit with providing analysis results via generating graphs exhibiting enriched GO terms as well as their relationships in the whole GO hierarchical tree. In addition, Database for Annotation, Visualization, and Integrated Discovery (DAVID, http://david.abcc.ncifcrf.gov) was also used to conduct GO terms functional analysis with displaying gene names for a give gene list [17]. The DEGs identified and sorted into hierarchical clusters by GOEAST were based on the cellular component, molecular function, and biological process using hypergeometric method. The probes on the microarray were considered as background, and *p* values of < 0.001 were considered to indicate statistical significance in both software analysis.

### Pathway enrichment analysis

Biological functions of the DEGs were further explored at the molecular level. Kyoto Encyclopedia of Genes and Genomes (KEGG, http://www.genome.jp/kegg/) pathway enrichment analysis was aimed to gene-related pathway annotations based on KEGG database. In the present study, cluster analysis of pathways was performed with a hypergeometric algorithm using WEB-based GEne SeT AnaLysis Toolkit (WebGestalt; http://www.webgestalt.org/) (*p* < 0.05), an important software tool designed for functional genomic, proteomic, and large-scale genetic studies from which large sets of genes are generated [18].

### Prediction of potential sites of miRNA that targeted by DEGs

The potential binding sites of miRNAs were predicted based on Molecular Signatures Database (MSigDB, http://www.broadinstitute.org/gsea/msigdb/index.jsp) [19], in which the set consisted of genes grouped by share short sequence motifs make it possible to predict the regulatory relationships between genes and putative miRNAs element. Enrichment analysis of the data set was performed using a hypergeometric test with Benjamini–Hochberg correction, and *p* < 0.05 was set as cut-off for significant miRNAs.

### Function analysis of targets of predicted miRNA

After obtaining the regulatory relationships between predicted miRNA and targeted DEGs, the functional enrichment analysis of targeted DEGs of putative miRNAs were performed by DAVID. *P* < 0.05 was used as threshold for significant results.

## Results

### Identification of DEGs

The gene expression profiles of the experimental (treated with estrogen) and control groups were analyzed using Bayes t-test [20] (Bayesian model corrected). Using *p* values of < 0.05 as the statistical significance threshold, a total of 400 gene probes, including 321 DEGs were identified (Additional file 1).

### PPI network construction and submodule analysis of PPI network

**Fig. 1:**
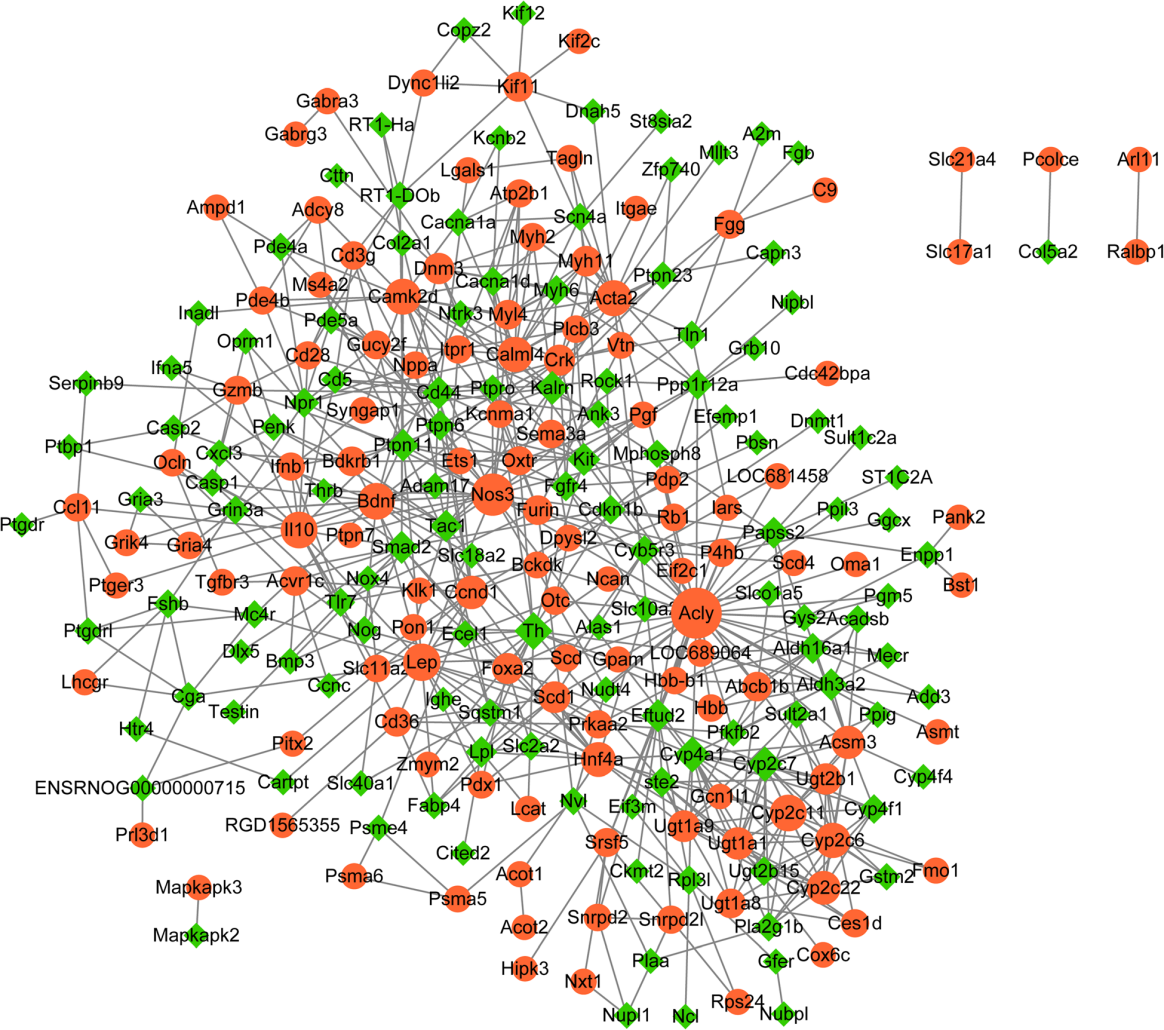
The PPI network for the DEGs. Node represents genes and edge connects the nodes to indicate interactions among them. The red circle node represents up-regulated DEGs, while the green rhombus node stands for down-regulated DEGs. The node size represents connectivity degree. PPI: protein–protein interaction; DEGs: differentially expressed genes

The PPI network comprised of 243 nodes and 590 interaction pairs (Fig. 1 and Additional file 2). The nodes like Acly, Nos3, Th, Lep, Bdnf and Cyp2c11 had higher connective degrees in this network (Additional file 3).

**Fig. 2:**
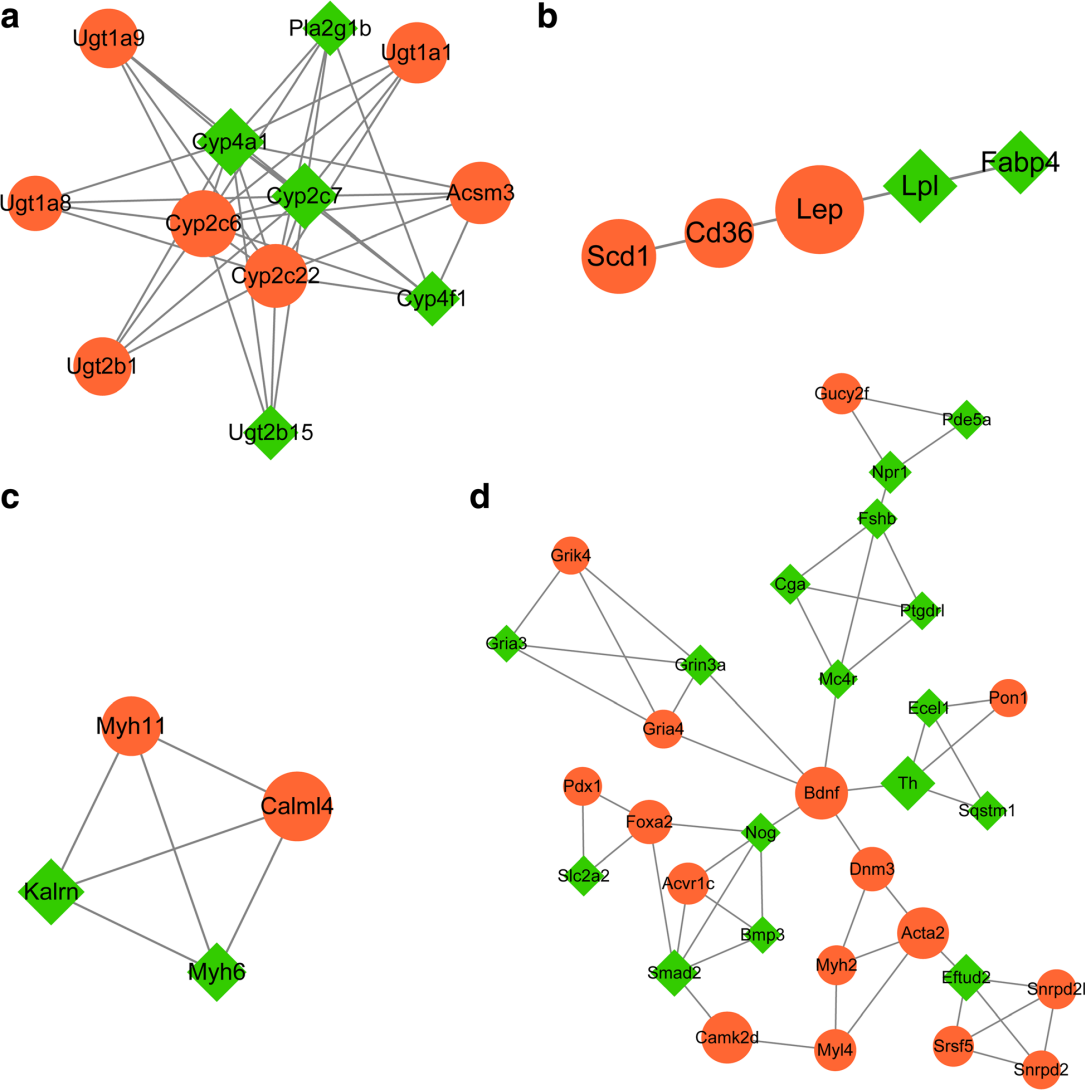
The results of submodule anlysis of PPI network. **a** The sub-network of module a; **b** The sub-network of module b; **c** The sub-network of module c; **d** The sub-network of module d. Node represents genes and edge connects the nodes to indicate interactions among them. The red circle node represents up-regulated genes, while the green rhombus node stands for down-regulated genes. The node size represents connectivity degree. PPI: protein–protein interaction

Based on aforementioned threshold value, four submodules were obtained from PPI network. Module a was consisted of 12 nodes with corresponding 40 interaction pairs, while a total of 5 nodes with corresponding 10 interaction pairs were included in module b. module c was consisted of 4 nodes and 6 interaction pairs, and 32 nodes and 52 interaction pairs were included in module d (Fig. 2 and Additional file 4). Most of above nodes with high degree in the PPI network were also highlighted in module a (eg., Cyp2c6, Cyp2c7 and Cyp2c22), module b (eg., Lep), module c (eg., Calml4 and Kalrn) and module d (eg., Th and Bdnf). Meanwhile, most of UDP Glucuronosyltransferase family members such as Ugt1a1, Ugt1a9, Ugt1a8, Ugt2b1 and Ugt2b15 were enriched in module a. Moreover, four glutamate receptors as Gria4, Gria3, Grin3a and Grik4 were highlighted in module d (Additional file 4).

### GO analysis of the DEGs

**Fig. 3:**
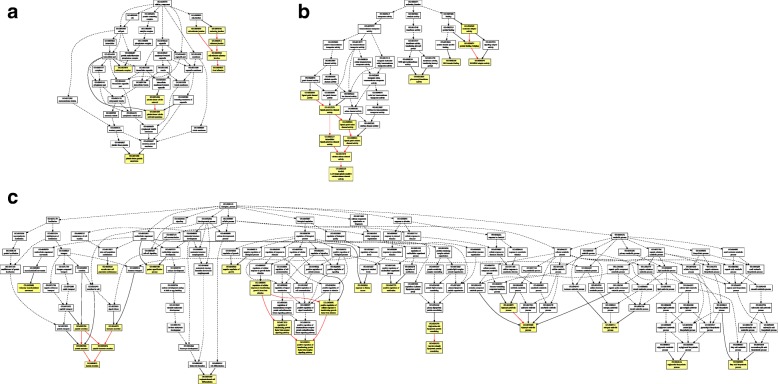
**a** The results of differentially expressed genes clustered based on cellular components. The yellow color represents the significantly enriched cellular component (the significance is positively related to the color). **b** The results of differentially expressed genes clustered based on molecular functions. The yellow color represents the significantly enriched molecular function (the significance is positively related to the color). **c** The results of differentially expressed genes clustered based on biological processes. The yellow color represents the significantly enriched biological process (the significance is positively related to the color)

The GO enrichment analysis was conducted by applying GOEAST. The clustering result of the DEGs based on cellular components is shown in Fig. 3a, the clustering results of the DEGs based on molecular functions is shown in Fig. 3b, and the clustering result of the DEGs based on biological processes is shown in Fig. 3c.

The result of the cellular component analysis indicated that the expression of genes related to calcineurin complex was significantly altered, which is consistent with variations in calcium channels based on the molecular function analysis. In addition, variation in the extracellular connection was also detected, which is critical for an extracellular signal response. Furthermore, variations were also identified in the platelet membrane and tubular network, which may be closely associated with the alleviation in estrogen levels in injured brain cells (Fig. 3a).

Notably, the molecular function enrichment analysis results indicated that the DEGs were mainly involved in the calcium channel, protein binding, and SH3/SH2-binding activity. The results also demonstrated that the intracellular signal pathways were altered after estrogen treatment. Moreover, the activity of UDP-glucuronosyl transferases was also significantly altered after estrogen treatment (Fig. 3b).

The biological process enrichment analysis results revealed that the TGF-β receptor signaling pathway, epidermal cell migration, insulin secretion, low-density lipoprotein reconstruction, trophectoderm differentiation, estrogen catabolism, benzidine metabolism, and triglyceride synthesis were significantly altered. Those pathways might be the potential molecular mechanisms for underlying cerebral injury (Fig. 3c).

Additionally, in order to display gene names with a give gene list for related go terms, the DAVID software were applied to conduct functional analysis. Under the threshold value of *p* < 0.001, the up-regulated DEGs were enriched in 25 GO terms, while the down-regulated DEGs were enriched in 18 GO terms (Additional file 5). Interestingly, both up-regulated and down-regulated DEGs were closely associated with anti-apoptosis (BDNF, ETS1, HIPK3, ERPINB9, SQSTM1, ADAM17, and CITED2) and response to endogenous stimulus (Table 1).

**Table 1:** The top 10 enriched Gene Ontology terms for down-regulated and up-regulated DEGs

	Category	Term	Count	PValue	Genes
	CC	GO:0044459~plasma membrane part	39	4.14E-07	OPRM1, CYB5R3, TLN1, RAB3C, RAB3D…
	BP	GO:0006916~anti-apoptosis	4	1.69E-06	SERPINB9, SQSTM1, ADAM17, CITED2
	CC	GO:0005886~plasma membrane	54	1.88E-06	P2RX1, MC4R, CACNA1D, SCN4A, CACNA1A…
Down-regulated DEGs	BP	GO:0010033~response to organic substance	27	2.00E-05	P2RX1, SLC18A2, MC4R, ADAM17, FABP4…
	CC	GO:0005924~cell-substrate adherens junction	8	3.96E-05	NOX4, OPRM1, TLN1, PGM5, CD44…
	CC	GO:0030055~cell-substrate junction	8	5.96E-05	NOX4, OPRM1, TLN1, PGM5, CD44…
	BP	GO:0007267~cell-cell signaling	14	1.17E-04	CGA, EDN3, FGFR4, RAB3C, TH…
	CC	GO:0009898~internal side of plasma membrane	10	1.55E-04	TH, KIT, VPS33B, NUPL1, ADD3…
	BP	GO:0003001~generation of a signal involved in cell-cell signaling	8	2.39E-04	CCKAR, CGA, EDN3, RAB3C, P2RX1…
	CC	GO:0005925~focal adhesion	7	2.51E-04	NOX4, OPRM1, TLN1, PGM5, CD44…
	BP	GO:0042493~response to drug	19	1.57E-08	BDNF, UGT1A9, UGT1A8, UGT1A7C, UGT1A3…
	BP	GO:0044093~positive regulation of molecular function	20	2.42E-06	DPDX1, IL10, CCND1, PSMA6, IFNB1,…
	BP	GO:0009725~response to hormone stimulus	20	2.63E-06	UGT1A6, UGT1A9, UGT1A8, UGT1A7C, UGT1A3…
UP-regulated DEGs	BP	GO:0009719~response to endogenous stimulus	20	1.40E-05	DLC1, ADCY8, NOS3, KCNMA1, LEP…
	BP	GO:0006631~fatty acid metabolic process	11	3.93E-05	SCD1, LEP, ACSM3, CD36, SCD…
	CC	GO:0005792~microsome	13	5.10E-05	SCD1, CYP2C6, P4HB, SCD, UGT2B1…
	CC	GO:0045177~apical part of cell	11	5.61E-05	OXTR, NOS3, KLK1, RGD1565355, CLCN5…
	CC	GO:0005829~cytosol	29	6.28E-05	TPN7, DNM3, LOC686737, RALBP1, VHL…
	CC	GO:0042598~vesicular fraction	13	6.82E-05	ITPR1, UGT1A6, UGT1A9, UGT1A8, CD36…
	BP	GO:0006916~anti-apoptosis	3	1.43E-04	BDNF, ETS1, HIPK3

*DEGs* differentially expressed genes, *BP* biological process, *CC* cellular component

### Biological pathways analysis

The biological pathways enrichment (*p* < 0.05) results demonstrated that several critical metabolic pathways, including starch and sucrose metabolism, retinal metabolism, vitamin C metabolism, and transformation between pentose and glucuronic acid, were significantly altered in the brain cells of estrogen-treated samples (Table 2 and Additional file 6). The results suggested that the protective effect of estrogen in cerebral ischemic injury is achieved by improvement in the metabolism in injured brain cells.

**Table 2:** The enriched KEGG pathways of differentially expressed genes

KEGG pathway	*P*- value
Starch and sucrose metabolism	0.0202
Retinol metabolism	0.0269
Ascorbate and aldarate metabolism	0.0269
Pentose and glucuronate interconversions	0.0454

### Potential regulatory miRNAs prediction

miRNA regulates the gene expression by controlling the stability of the target RNA. Therefore, the potential regulatory miRNAs were identified based on the DEG sequences. The only five significant miRNAs were identified, and the target-binding sites and targets genes of those miRNAs are listed in Table 3: rno_ATGCTGG (MIR-338), rno_TTTGTAG (MIR-520D), rno_TCTATGA (MIR-376A, MIR-376B), and rno_CTCCAAG (MIR-432) (Additional file 7).

**Table 3:** The potential regulatory microRNAs

Targeted-sequence	Potential micro-RNA	P-value	Targeted genes
rno_ATGCTGG	MIR-338	0.0118	Adam17, Kcnma1, Ets1, Ccnd1, Syt8, Atp2b1
rno_TTTGTAG	MIR-520D	0.0143	Sfrs5, Ets1, Ccnd1, Kif11, Eif2c1, Atp2b1, Kcnma1, NIPBL, Cited2, Crhbp, Scd
rno_TCTATGA	MIR-376A, MIR-376B	0.0062	Dlx5, NIPBL, Capn3, Nupl1, Grin3a, Adnp
rno_CTCCAAG	MIR-432	0.0142	Capn3, Furin, Pde4a, Tln1, Eif2c1

### Function analysis of targets of predicted miRNA

With using aforementioned threshold value and method, the targets of four miRNAs were enriched in several GO terms (Table 4). However, no significant KEGG pathways were obtained. As shown in Table 4, the targets of MIR-338 were closely with 17 biological process (eg., response to endogenous stimulus, positive regulation of cell motion and regulation of homeostatic process), and 1 molecular function as PDZ domain binding. Meanwhile, the targets of MIR520D were strongly associated with positive regulation of specific transcription from RNA polymerase II promoter, cell cycle, and regulation of action potential in neuron. In addition, the targets of MIR-376A and MIR-376B were only involved in cell soma.

**Table 4:** The results of functional analysis of targets of predicted microRNAs

	Category	Term	Count	PValue	Genes
	BP	GO:0045737~positive regulation of cyclin-dependent protein kinase activity	2	0.002892	CCND1, ADAM17
	BP	GO:0000079~regulation of cyclin-dependent protein kinase activity	2	0.009065	CCND1, ADAM17
	BP	GO:0000082~G1/S transition of mitotic cell cycle	2	0.015209	CCND1, ADAM17
	BP	GO:0009719~response to endogenous stimulus	3	0.020374	KCNMA1, CCND1, ADAM17
	BP	GO:0045787~positive regulation of cell cycle	2	0.023758	CCND1, ADAM17
	BP	GO:0051592~response to calcium ion	2	0.026189	KCNMA1, CCND1
	BP	GO:0051329~interphase of mitotic cell cycle	2	0.026594	CCND1, ADAM17
MIR-338	MF	GO:0030165~PDZ domain binding	2	0.026878	ATP2B1, ADAM17
	BP	GO:0051325~interphase	2	0.027403	CCND1, ADAM17
	BP	GO:0042981~regulation of apoptosis	3	0.027951	KCNMA1, ETS1, ADAM17
	BP	GO:0043067~regulation of programmed cell death	3	0.028656	KCNMA1, ETS1, ADAM17
	BP	GO:0010941~regulation of cell death	3	0.028892	KCNMA1, ETS1, ADAM17
	BP	GO:0001934~positive regulation of protein amino acid phosphorylation	2	0.038676	CCND1, ADAM17
	BP	GO:0010604~positive regulation of macromolecule metabolic process	3	0.040483	CCND1, ETS1, ADAM17
	BP	GO:0051272~positive regulation of cell motion	2	0.040678	ETS1, ADAM17
	BP	GO:0042327~positive regulation of phosphorylation	2	0.042677	CCND1, ADAM17
	BP	GO:0045937~positive regulation of phosphate metabolic process	2	0.043874	CCND1, ADAM17
	BP	GO:0032844~regulation of homeostatic process	2	0.045867	ETS1, ADAM17
	BP	GO:0051726~regulation of cell cycle	3	0.013609	CCND1, SFRS5, CITED2
MIR520D	BP	GO:0010552~positive regulation of specific transcription from RNA polymerase II promoter	2	0.037336	ETS1, CITED2
	BP	GO:0045787~positive regulation of cell cycle	2	0.042363	CCND1, CITED2
	BP	GO:0051592~response to calcium ion	2	0.046653	KCNMA1, CCND1
	BP	GO:0019228~regulation of action potential in neuron	2	0.048792	KCNMA1, SCD
MIR-376A, MIR-376B	CC	GO:0043025~cell soma	2	0.046407	ADNP, GRIN3A

*BP* biological process, *CC* cellular component

## Discussion

The present study systemically analyzed the gene expression profiles of estrogen-treated ischemic cells and identified a total of 321 DEGs. The GO analysis results indicated that the expression of genes related to Ca^2+^ channel and calcineurin complex was significantly altered. The activation of Ca^2+^ channel in cerebral ischemic injury has also been studied previously [4]. Because of the energy cut-off, the presynaptic voltage-dependent Ca^2+^ channel is activated due to damaged membrane potential [4].

The extracellular Ca^2+^ is essentially required for the expression of glutamate-induced prokineticin 2, an endangering mediator of cerebral ischemic injury. Furthermore, calcium dysregulation is one of the primary instigators, and the increase in calcium influx and damage of calcium extrusion between the membrane leads to impaired neuronal function in cerebral ischemic injury [21]. The investigation of the protective mechanism of Cav2.1 channel in ischemic models has indicated its potential application in preventing ischemic injury [21]. Moreover, the number of genes related to metabolism was significantly altered in estrogen-treated cells. The direct damage caused by stroke is the reduction in the energy supply, including reduction in oxygen and glucose levels. Consequently, homeostasis is dysregulated. It has been suggested that resveratrol can enhance the neuroprotective effect, which can further improve brain metabolism [22].

One study has shown that estrogen can exert protective effects via mitochondrial mechanisms [23]. In a recent investigation, the production of mitochondrial reactive oxygen species was suppressed and mitochondrial efficiency was significantly enhanced in cerebral blood vessels after estrogen treatment [24]. Reportedly, mitochondrial ATP levels could be improved and cell death could be prevented by an endoplasmic reticulum-mediated mechanism in in vitro ischemic model treated with 17β-estradiol [25], indicating that metabolic pathways are closely associated with cerebral ischemic injury.

The TGF-β receptor signaling pathway has also been shown to play an important role in brain ischemic injury. In one study, TGF-β gene expression was significantly upregulated in ischemic cells compared with that in the normal cells [26]. Several studies have demonstrated that TGF-β can act as a neuroprotective factor in the pathogenesis of stroke. Moreover, in the rodent models of cerebral ischemia, the TGF-β mRNA expression is increased following an ischemic event [27]. Ruocco et al. have suggested that the administration of TGF-β-blocking agent can significantly increase excitotoxic lesions after cerebral ischemia [28] and indicated that TGF-β can diminish ischemia-induced endothelial dysfunction [28]. However, the molecular mechanism of TGF-β in the cerebral ischemia is largely unknown.

In the submodule analysis, four glutamate receptors associated DEGs (Gria4, Gria3, Grin3a and Grik4) were enriched in module d. It is well known that the abnormal activation of glutamate receptors in hypoxia-ischemia conditions may induce excitotoxicity via increasing Ca^2+^, Na^+^, and Zn^2+^ internal flow during ischemic stroke patients [29]. Akins et al have suggested glutamate AMPA receptor antagonist is an effect treatment for ischaemic stroke [30]. In addition, it has been reported estradiol can reduce the level of the Type I metabotropic glutamate receptors and completely prevent cell death to alleviate excitotoxic brain damage in the hippocampal neurons [31]. As expected, our study have identified the expression of four glutamate receptors associated genes as *Gria4*, *Gria3*, *Grin3a* and *Grik4* were significantly altered, which edited Glutamate Ionotropic Receptor AMPA Type Subunit or NMDA Type Subunit. Thus, estrogen may alleviate excitotoxic brain damage via regulating several glutamate receptors types. Notably, we predicted that estrogen treatment can induce the upregulation of *BDNF* and downregulation of *ADAM17* in MCAO rat, which were associated with the GO terms of anti-apoptosis. Reportedly, estrogen can exert neuroprotection function in MCAO rat by inhibiting Fas-mediated apoptosis [32]. The up-regulated BDNF may inhibit cell apoptosis in the cerebral ischemia rat [33]. Additionally, the activation of ADAM17 activity in neutrophils may induce the neutrophil apoptosis [34]. Therefore, we speculated that estrogen was involved in *BDNF* and *ADAM17* induced anti-apoptosis in MCAO rat.

In addition, the metabolism-related KEGG pathways, including starch and sucrose metabolism and retinol metabolism pathways, were also significantly altered. The genes, such as *Gys2* and *Ugt1a2*, of the starch and sucrose metabolism pathway mainly participate in glycogen synthesis and transfer of the glucuronic acid component of UDP-glucuronic acid. However, in cerebral ischemic injury, the oxygen supply is cut off and the glucose consumption is blocked. The *Gys2* and *Ugtla2* genes can regulate the glycogen/glucose level and promote the storage of glycogen, suggesting that estrogen increases the expression of starch and sucrose metabolism-related genes and reduces the effects of ischemic injury.

Further, the potential miRNA target sites were predicted. Of the screened miRNA target sites, the majority were related to ATP dephosphorylation, Ca^2+^ transporting, and calcium-activated channels, such as *Kcnma1* and *Atp2b1*. Their functions have been demonstrated to be associated with cerebral ischemic injury [4]. In addition, our results indicated that MIR-338 may play an important role on the neuroprotection in cerebral ischemic induced by estrogen via regulating cell cycle and cell motion associated genes (eg., *CCND1*), respectively. Todd E et al. have reported that suppression of cell cycle associated gene *CCND1* was closely involved in contralateral to traumatic brain injury [35]. Moreover, it has reported that miR-338-3p is required for liver cell proliferation via regulating Cyclin D1 expression [36]. Thus, the estrogen may have function role on regulating cell cycle in cerebral ischemic mediated by miR-338-3p and Cyclin D1. However, some of the regulatory miRNAs, such as MIR-376A, have not been reported previously. *ADNP* and *GRIN3A* as DEGs in estrogen-treated MCAO rat were predicted to be targets of MIR-376A, and were involved in cell soma. Those results provide novel mechanisms of estrogen in cerebral ischemic injury, but it still need future investigation.

## Conclusions

In summary, the analysis of gene expression in estrogen-treated cerebral ischemic injury samples revealed some functionally significant DEGs and several new target sites, which may serve as potential therapeutic targets for the effective treatment of cerebral ischemic injury.

## Additional files

Additional file 1: Differentially expressed genes identified by RNA-seq. The excel lists a total of 400 probes with the expression level changed, and those probes involved 321 differentially expressed genes between estrogen-treated intraluminal middle cerebral artery occlusion (MCAO) rat group and untreated MCAO rat group (*p*-value < 0.05). (XLSX 42 kb)

Additional file 2: The interaction pairs of nodes in protein-protein interaction network. This excel presents 243 nodes and 590 interaction pairs (combined score > 0.4) in Fig. 1. In addition, it contains the combined score values of these 590 interaction pairs. (XLSX 61 kb)

Additional file 3: The degrees of nodes in protein-protein interaction network. The excel describes the up/down-regulated status and degrees of 243 nodes in the PPI network. A total of 119 nodes were up-regulated genes and 124 nodes were down-regulated genes. The nodes degrees were ranged from 37 to 1. (XLSX 227 kb)

Additional file 4: The degrees of nodes in Modules. The excel describes the up/down-regulated status and degrees of nodes in modules a, b c and d. Module a consists of 7 up-regulated and 5 down-regulated genes, and the degrees of those nodes were ranged from 16 to 7. There were 3 up-regulated and 2 down-regulated genes in module b, and the degrees of nodes were ranged from 18 (Lep) to 4 (Fabp4). Additionally, two upregulated and two downregulated comprised of module c, in which the degrees of nodes were ranged from 15 to 8. In module d, the degrees of nodes were ranged from 19 (Th) to 3(Grik4). (XLSX 222 kb)

Additional file 5: GO items enriched by up-regulated and down-regulated differentially expressed genes. The excel provides the following information in detail, including the names of 25 GO terms enriched by up-regulated genes and 18 GO terms enriched by down-regulated genes, and category, count, *p* value and adjusted *p* values for each GO term, as well as the genes list that enriched in GO term. (XLSX 16 kb)

Additional file 6: KEGG pathways enriched by differentially expressed genes. The results for each enriched KEGG pathway are listed in this excel. For each KEGG pathway, the information including KEGG pathway name, corresponding KEGG ID, number of genes in the gene set and also in the category, p value from hypergeometric test, p value adjusted by the multiple test adjustment, as well as genes in the pathway are listed. (XLSX 14 kb)

Additional file 7: The potential regulatory microRNAs. The results for each enriched gene set of predicted microRNA are listed in this excel. For each predicted microRNA, the information including the microRNA name, corresponding Gene Set ID, number of target genes of each microRNA, *p* value from hypergeometric test, p value adjusted by the multiple test adjustment, as well as target genes of each microRNA are listed. (XLSX 18 kb)

## Abbreviations

DEGs: Differentially expressed genes
GEO: Gene Expression Omnibus
GO: Gene Otology
GOEAST: Gene Ontology Enrichment Analysis Software Toolkit
KEGG: Kyoto Encyclopedia of Genes and Genomes
